# Dietary Supplementation of Postbiotics Mitigates Adverse Impacts of Heat Stress on Antioxidant Enzyme Activity, Total Antioxidant, Lipid Peroxidation, Physiological Stress Indicators, Lipid Profile and Meat Quality in Broilers

**DOI:** 10.3390/ani10060982

**Published:** 2020-06-05

**Authors:** Ali Merzza Humam, Teck Chwen Loh, Hooi Ling Foo, Wan Ibrahim Izuddin, Elmutaz Atta Awad, Zulkifli Idrus, Anjas Asmara Samsudin, Noordin Mohamed Mustapha

**Affiliations:** 1 Department of Animal Science, Faculty of Agriculture, University Putra Malaysia, UPM Serdang 43400, Selangor, Malaysia; humamalimerzza@gmail.com (A.M.H.); wanahmadizuddin@gmail.com (W.I.I.); zulidrus@upm.edu.my (Z.I.); anjas@upm.edu.my (A.A.S.); 2 Department of Animal Production, Faculty of Agricultural Engineering Sciences, University of Baghdad, Baghdad 10071, Iraq; 3 Institutes of Tropical Agriculture and Food Security, University Putra Malaysia, UPM Serdang 43400, Selangor, Malaysia; motazata83@gmail.com; 4 Department of Bioprocess Technology, Faculty of Biotechnology and Biomolecular Science, University Putra Malaysia, UPM Serdang 43400, Selangor, Malaysia; 5 Institute of Bioscience, University Putra Malaysia, UPM Serdang 43400, Selangor, Malaysia; 6 Preclinical Department, Faculty of Veterinary Medicine, University Malaysia Kelantan, Pengkalan Chepa 16100, Kelantan, Malaysia; 7 Department of Veterinary Pathology & Microbiology, Faculty of Veterinary Medicine, University Putra Malaysia, UPM Serdang 43400, Selangor, Malaysia; noordinmm@upm.edu.my

**Keywords:** broilers, heat stress, antibiotic, postbiotic, antioxidant enzyme activity, lipid peroxidation, acute phase proteins, heat shock protein 70, lipid profile, meat quality

## Abstract

**Simple Summary:**

To mitigate the adverse impacts of stressful environmental conditions on poultry and to promote the animal’s health and growth performance, antibiotics at sub-therapeutic doses have been added to poultry diets as growth promoters. Nevertheless, the improper and overuse of antibiotics as feed additives have played a major role in the emergence of antibiotic-resistant bacteria and increased levels of antibiotic residues in animal products, which have disastrous effects on the health of both animals and humans. Postbiotics, used as dietary additives for livestock, could be potential alternatives to antibiotics. Postbiotics produced from the probiotic *Lactobacillus plantarum* have been the subject of several recent kinds of research. However, the researchers have very rarely considered the effect of postbiotics on the broilers under heat stress.

**Abstract:**

The purpose of this work was to evaluate the impacts of feeding different postbiotics on oxidative stress markers, physiological stress indicators, lipid profile and meat quality in heat-stressed broilers. A total of 252 male Cobb 500 (22-day-old) were fed with 1 of 6 diets: A basal diet without any supplementation as negative control (NC); basal diet + 0.02% oxytetracycline served as positive control (PC); basal diet + 0.02% ascorbic acid (AA); or the basal diet diet + 0.3% of RI11, RS5 or UL4 postbiotics. Postbiotics supplementation, especially RI11 increased plasma activity of total-antioxidant capacity (T-AOC), catalase (CAT) and glutathione (GSH), and decreased alpha-1-acid-glycoprotein (α1-AGP) and ceruloplasmin (CPN) compared to NC and PC groups. Meat malondialdehyde (MDA) was lower in the postbiotic groups than the NC, PC and AA groups. Plasma corticosterone, heat shock protein70 (HSP70) and high density lipoprotein (HDL) were not affected by dietary treatments. Postbiotics decreased plasma cholesterol concentration compared to other groups, and plasma triglyceride and very low density lipoprotein (VLDL) compared to the NC group. Postbiotics increased breast meat pH, and decreased shear force and lightness (L*) compared to NC and PC groups. The drip loss, cooking loss and yellowness (b*) were lower in postbiotics groups compared to other groups. In conclusion, postbiotics particularly RI11 could be used as an alternative to antibiotics and natural sources of antioxidants for heat-stressed broilers.

## 1. Introduction

Environmental stressors such as disease and heat stress are the major problems faced by global poultry production, having negative influences on animal physiology, behaviour, health and productive performance, causing tremendous economic losses [1,2,3,4,5]. They can adversely affect the biological macromolecules such as proteins, lipids, carbohydrates and DNA by the generation and accumulation of reactive oxygen species (ROS) and free radicals in the cells while performing their normal metabolic functions [6,7,8], resulting in cell damage and the appearance of pathological symptoms [9,10]. To mitigate the adverse impacts of stressful environmental conditions on poultry and to promote the animal’s health and growth performance, antibiotics at sub-therapeutic doses have been added to poultry diets as growth promoters [11,12,13]. However, the improper and overuse of antibiotics as feed additives have played a major role in the emergence of antibiotic-resistant bacteria and increased the levels of antibiotic residues in animal products, which have disastrous effects on the health of both animals and humans [14,15]. Thus, the use of antibiotics in farming livestock has been prohibited in the EU [16,17]. Ascorbic acid is a natural antioxidant and health-promoting agent that has great potential to substitute antibiotics as growth promoters in fighting bacterial infections [18,19], and have been thought to be beneficial for heat-stressed broiler chickens. Dietary supplementation of ascorbic acid has the advantage of compensating for incompetent biosynthesis of ascorbic acid and has the potential to ameliorate the harmful effects of hot climate in broiler chickens [20]. Data from several studies suggest that ascorbic acid supplementation may compensate for the reduction in growth rate and feed intake [21,22], improve overall growth performance, antioxidant status and meat quality, and reduce serum concentrations of corticosterone, acute phase proteins, cholesterol and lipid oxidation [23,24].

Using postbiotics as dietary additives for livestock and potential alternatives to antibiotics, postbiotics produced from the probiotic *Lactobacillus plantarum* have been the subject of several recent kinds of research. The mechanism of action of postbiotics is not different from that of probiotics, owing to the fact that the same secondary metabolites from probiotics are present in the postbiotics, but not the living cells [25]. Postbiotics contain several antimicrobial components including bacteriocins and organic acids, which can minimise the pH of the gut and prevent the proliferation of pathogens in both the feed and animal gut [26]. Recent evidence suggests that the postbiotics produced by *L. plantarum* strains have an inhibitory effect on several gut pathogens, such as vancomycin-resistant Enterococci, *Listeria monocytogenes, Salmonella typhimurium* and *Escherichia coli* [27,28,29,30]. It has recently been observed that the dietary supplementation of postbiotics promoted the health and growth performance in broilers [31,32,33], layers [34,35] and piglets [36,37]. More recently, postbiotics have been revealed to enhance the growth performance, rumen fermentation, immune status and gastrointestinal health in small ruminants [38,39]. Under normal environmental temperature, dietary supplementation of postbiotics improved health and growth performance of broiler chickens by promoting their immune status, growth genes expression and gut health, as their supplementation significantly improved the intestinal villus, decreased the population of Enterobacteriaceae and faecal pH, and increased the population of lactic acid bacteria [25,31,32,33,40]. Moreover, improvements in broiler meat quality and reduction in plasma cholesterol were observed with dietary supplementation of postbiotics in broilers [35,40,41,42]. Our recent findings from a companion study [43] revealed that dietary supplementation of postbiotics produced from *L. plantarum* increased body weight, body weight gain, feed conversion ratio (FCR), intestinal villus height, immune response, insulin-like growth factor 1 (IGF-1) and growth hormone receptor (GHR) mRNA expression, caecum non-pathogenic bacteria population, and reduced Enterobacteriaceae and *E. coli* population in heat-stressed broilers.

Aside from developing a healthy gut and promoting growth performance, a preliminary study from this laboratory revealed that the postbiotics produced by *L. plantarum* have high antioxidant activities [44]. Similarly, bacterial cultures of *L. plantarum* were reported to exhibit high antioxidative activities [45,46]. Whilst considerable research has investigated the beneficial impacts of postbiotics on broiler chickens under normal temperature, there is still a scarcity of information on their impacts on heat-stressed broilers. Therefore, the outlook of this work was to examine the impacts of feeding three different postbiotics on antioxidant enzyme activity, total antioxidant, lipid peroxidation, heat shock protein 70, acute phase proteins, lipid profile and meat quality in heat-stressed broiler chickens.

## 2. Materials and Methods 

### 2.1. Postbiotics Production

The three different *Lactobacillus plantarum* strains, RS5, RI11 and UL4 were procured from the Industrial Biotechnology Laboratory, Faculty of Biotechnology and Biomolecular Sciences, University Putra Malaysia. The DNA was sequenced by Moghadam et al. [47] who differentiated between the *L. plantarum* strains, while their ability for producing different amino acids was recently determined by Lim et al. [48]. The three different cultures were preserved by the revival of cultures following Foo et al.’s procedure [49]. The cultures were kept at −20 °C in De Man, Rogosa and Sharpe MRS medium (Merck, Darmstadt, Germany) with 20% (*v*/*v*) glycerol.

A volume of 100 µL from each stock culture was activated in 10 mL MRS broth, incubated at 30 °C for 48 h and sub-cultured in the same media for another 24 h. The activated cultures were spread onto a plate and incubated at 30 °C for 48 h. An individual colony was picked from the plate, inoculated twice into MRS broth (10 mL) and incubated at 30 °C for 48 h and 24 h. Active cells of *L. plantarum* (RI11, UL4 and RS5) were first washed using a 0.85% (*w*/*v*) NaCl (Merck, Darmstadt, Germany) sterile solution, then adjusted to 10^9^ CFU/mL and used as an inoculum. For preparing the working cultures of the three *L. plantarum* strains (RS5, UL4 and RI11), 10% (*v*/*w*) 10^9^ CFU/mL active bacterial cells were inoculated into MRS media, incubated for 10 h at 30 °C, and centrifuged at 10,000× *g* for 15 min at 4 °C. Cell-free supernatant (CFS) was filtered using a 0.22 µm cellulose acetate membrane (Sartorius Minisart, Gottingen, Germany) following the procedure outlined in detail by Loh et al. [50]. The harvested CFS (postbiotic) was kept at −20 °C until further analysis.

### 2.2. Ethical Note, Birds, Experimental Design and Housing

The feeding trial was performed at the research facilities of the Institute of Tropical Agriculture and Food Security (ITAFoS), University Putra Malaysia. The experiment was conducted in conferment with the approved guidelines by the Animal Ethics Committee of the University Putra Malaysia (protocol no. UPM/ACUC/AUP-R085/2018), which ascertains that the use and care of research animals are ethical and humane. Two hundred and fifty-two Cobb 500 male chicks (one-day-old) were supplied by a local hatchery. The chicks were housed in wire-floor cages placed in two identical rooms. The rooms were environmentally controlled with each of the two measuring 9.1 × randomised 3.8 × 2.3 m, length × width × height, whereas measurement of each cage was 120 (length) × 120 (width) × 45 (height) cm. The birds were reared following the management recommendations of Cobb 500 from 1 to 21 days of age (starter period). The chickens in the two rooms were maintained at the recommended temperature of 32 ± 1 °C on the first day, and gradually reduced to around 24 ± 1 °C by 21 days of age. During the finisher period (day 22 to day 42), the birds were divided into 6 treatment groups, 7 replicates per group with 6 chicks in each replicate. The birds were offered 1 of 6 diets: (1) A basal diet without any supplementation as negative control (NC); (2) basal diet + 0.02% (*w*/*w*) oxytetracycline as positive control (PC); (3) basal diet + 0.02% (*w*/*w*) ascorbic acid as antioxidant control (AA); or basal diet + 0.3% (*v*/*w*) of (4) RI11, (5) RS5 or (6) UL4 postbiotics. The basal diets were formulated using FeedLIVE software Version 1.52 (Live Informatics Company Ltd., Nonthaburi, Thailand) [43] following the nutrient specifications of the Cobb 500 Nutrition Guide. From day 22 to day 42, broilers were subjected to a high temperature for 3 h per day from 11:00 am to 2:00 pm at 36 ± 1 °C. It took approximately 45 min for the temperature to escalate from 24 to 36 °C. Nonetheless, it took 1 h and 30 min for the temperature to decline from 36 to 24 °C. The management and environmental conditions of this current experiment are described in our recently published companion study [43].

### 2.3. Samples Collection

At 42 days of age, around 2 h and 45 min after the daily heat stress, 2 chickens from each cage (14 chickens per treatment group) were selected at random and slaughtered following the Halal practice, as recommended by the Malaysian Standard [51]. Blood was collected at exsanguination into BD Vacutainer^®^ EDTA blood tubes (New Jersey, BD, USA) and kept on ice. Upon centrifugation at 3500× *g* for 15 min at 4 °C, harvested plasma samples were (1.5 mL microcentrifuge tubes) stored at −80 °C for later determination of total antioxidant capacity (T-AOC), glutathione peroxidase (GPx), superoxide dismutase (SOD), catalase (CAT), glutathione (GSH), corticosterone (CORT), ceruloplasmin (CPN), alpha 1-acid glycoprotein (α1-AGP), heat shock protein 70 (HSP70) and lipid profiles. Whole breast muscle was collected for the meat quality determination. The thigh muscle was collected for lipid peroxidation measurement.

### 2.4. Plasma Antioxidant Enzymes Biomarkers

#### 2.4.1. Total Antioxidant Capacity

Total antioxidant capacity (T-AOC) was measured by a colourimetric method (ABTS) using a commercial total antioxidant capacity assay kit (Elabscience, E-BC-K219, Houston, TX, USA) according to the manufacturer’s protocol. The principle of this method, to determine the T-AOC from plasma, is that ABTS is oxidised to green ABTS^+^ by appropriate oxidant, which can be inhibited in the presence of antioxidants. Briefly, 10 μL of plasma sample was loaded into a microplate well and mixed with 20 μL of application solution. Then, 170 μL of ABTS working solution was added into each well, and incubated at room temperature for 6 min. Finally, the absorbance of the colour was measured at 405 nm by a microplate reader (Multiskan GO, Thermo Scientific, Waltham, MA, USA). Blank contains distilled water plus application and working solutions were abstracted from samples and standard absorbance. The trolox solution was diluted with distilled water to prepare serial concentration (0.1, 0.2, 0.4, 0.8, and 1.0 mM) and the standard curve was plotted to determine T-AOC concentration in plasma.

#### 2.4.2. Superoxide Dismutase Activity

Superoxide dismutase (SOD) assays were carried out using EnzyChrom™ Superoxide Dismutase Assay Kit (ESOD-100, BioAssay Systems, Hayward, CA, USA) based on the protocol provided by the manufacturer. The detection range of the kit was 0.05–3 U/mL SOD. The test depended on the addition of xanthine oxidase to the sample as a source of superoxide, and this superoxide reacted with a specific dye to form a coloured product. Based on the activity of SOD in the sample, which acted as a superoxide scavenger, the superoxide was reduced, and then the intensity of colour was decreased. The activity of SOD was determined by measuring the colour intensity at 440 nm using a Multiskan GO microplate reader (Thermo Scientific, Waltham, MA, USA). The concentration of SOD in the sample was quantified using standard curve of known concentration of SOD.

#### 2.4.3. Catalase Activity

Catalase (CAT) activity was measured from plasma using the EnzyChrom^TM^ catalase assay kit (ECAT-100, BioAssay Systems, Hayward, CA, USA), according to the manufacturer’s instructions. The detection range of the kit was 0.2–5 U/L CAT. The test depends on the degradation of H_2_O_2_ using redox dye. After the preparation of the assay, 10 µL of the sample, positive control and assay buffer as blank plus 90 µL of substrate buffer (50 µM) were loaded into the micro-plate well, then the plate was shaken and incubated at room temperature for 30 min. During the incubation time, the standard curve was prepared by mixing 40 µL of the 4.8 mM H_2_O_2_ reagent with 440 µL of distilled water in the serial concentration, then 10 µL of the standard solution with 90 µL of assay buffer were placed into standard wells. After incubation, 100 µL of detection reagent was combined in each well and incubated for 10 min at room temperature. Finally, the optical density of CAT was read at 570 nm using a microplate reader (Multiskan GO, Thermo Scientific, Waltham, MA, USA). The standard curve was used to calculate the CAT activity in the plasma samples 

#### 2.4.4. Glutathione Peroxidase Activity

Glutathione peroxidase (GPx) activity was analysed in plasma samples using the EnzyChrom^TM^ Glutathione Peroxidase Assay Kit (EGPx-100, BioAssay Systems, Hayward, CA, USA), which directly measures the consumption of NADPH in the enzyme-coupled reactions. The assay was carried out as recommended in the manufacturer’s protocol. The detection range of the kit was 40–800 U/L GPx. Approximately, 10 µL of the sample plus 90 µL of working reagent (80 µL assay buffer, 5 µL glutathione, 3 µL NADPH (35 mM), and 2 µL gr enzyme) were loaded into the microplate well and the plate was tapped to mix. Next, 100 µL of substrate solution was added to each sample and control well. The optical density of the samples and standards were measured immediately at time zero (OD0), and again at 4 min (OD4). The absorbance of the GPx activity was recorded at 340 nm using a microplate reader (Multiskan GO, Thermo Scientific, Waltham, MA, USA). The NADPH standards were used to plot the standard curve. The standard curve was used to calculate the GPx activity in the plasma samples. 

#### 2.4.5. Glutathione Activity

Glutathione (GSH) activity was measured in plasma using QuantiChrom^TM^ Glutathione Assay Kit (DIGT-250, BioAssay Systems, Hayward, CA, USA) following the manufacturer’s protocol. The principle of the assay depended on the reaction of 5,5’-dithiobis-2-nitrobenzoic acid with reduced glutathione to form a yellow product. Briefly, 120 µL of 20-fold diluted sample was mixed with 120 µL of reagent A into 1.5 mL tube, centrifuged at 14,000 rpm for 5 min and 200 µL of supernatant was transferred into the microplate well. Then, 100 µL of reagent B was added to each well of samples, the plate was tapped for mixing, and incubated for 25 min at room temperature. Next, 400 µL of the calibrator was mixed in serial dilution with distilled water into separate wells as the standard and 300 µL of distilled water was pipetted into a separate well as a blank. After incubation, the absorbance was read at 412 nm using a microplate reader (Multiskan GO, Thermo Scientific, Waltham, MA, USA). The GSH concentration in the plasma was calculated using the standard curve of glutathione.

### 2.5. Plasma Lipid Profile 

Plasma total cholesterol (TCHOL), high density lipoprotein (HDL) and triglyceride (TG) were analysed using an automatic analyser 902 (Hitachi, Munich, Germany), while low-density lipoprotein (LDL) and very-low-density lipoprotein (VLDL) levels were estimated with the protocol described by DeLong et al. [52]. The level of VLDL was calculated by dividing plasma TG by five. The LDL level was obtained using the following equation:

LDL − C (mmol/L) = TC − HDL − C − TG/2.2(1)

### 2.6. Lipid Peroxidation

Thiobarbituric acid reactive substances (TBARS) in the thigh meat were measured with the method described by Lynch and Frei [53] and modified by Mercier et al. [54]. About 1 g of meat sample was homogenised in 4 mL 0.15 M KCl + 0.1 mM BHT for 1 min at 6000 rpm with Ultraturrax homogenizer. Two hundred microliters of the homogenised sample was mixed with a solution (TBARS solution), heated in a water bath set at 95 °C for 1 h, for a pink colour to develop. The formation of a pink coloured sample mixture is the result of the reaction of TBARS end products (malondialdehyde) with thiobarbituric acid (TBA). The following step was the cooling of the sample under tap water for 5 min. Three millilitres of n-butyl alcohol was then added to the extracts and homogenised. The next step was the centrifugation of the mixture for 10 min at 5000 rpm. The absorbance of the supernatant was read with a spectrophotometer (Secomam, Domont, France) at 532 nm wavelengths against n-butyl alcohol as a blank. TBARS concentration of samples was determined from a standard curve of 1, 1,3,3-tetraethoxypropane and calculated as µg malondialdehyde (MDA)/g meat.

### 2.7. Plasma Corticosterone 

The corticosterone (CORT) plasma concentration was measured using a CORT ELISA kit specific to chickens (QAYEE-BIO, Shanghai, China) in accordance with the instructions of the manufacturer. Fifty microliters of appropriately diluted samples and standard were loaded in duplicate into predetermined microplate wells, mixed with 50 μL of horseradish peroxidase solution, gently shaken and incubated at 37 °C for 60 min. Then, the contents of the wells were discarded and washed using washing solution. The last step was repeated five times. Then, 50 μL of chromogen solution A and 50 μL of chromogen solution B (of each) were pipetted into each well and incubated for 10 min at 37 °C while protected from light. After the incubation, 50 μL of a stop solution was pipetted into each well and the absorbance was immediately measured using a BioTek™ ELx800™ microplate reader (BioTek, Winooski, VT, USA) at 450 nm. A standard diluent (without horseradish peroxidase (HRP) or sample) was used as a blank, which was later subtracted from the absorbance of standards and samples. CORT concentration in plasma was calculated using an equation generated from the slope of the standard curve.

### 2.8. Heat Shock Protein 70 (HSP70)

The heat shock protein 70 (HSP70) plasma concentration was measured using chicken HSP70 ELISA kits (QAYEE-BIO, Shanghai, China) based on the manufacturer’s instructions. Except for the differences in assay standard, the principle, materials and steps used in this assay were similar to those described for plasma corticosterone determination (Section 2.7). HSP70 concentration in plasma was calculated using an equation generated from the slope of the standard curve.

### 2.9. Plasma Acute Phase Proteins

#### 2.9.1. α1-Acid Glycoprotein (α1-AGP)

The α1-AGP plasma concentration was measured using a commercial ELISA kit specific to chicken (QAYEE-BIO, Shanghai, China) following the manufacturer’s protocol. Except for the differences in assay standard, the principle, materials and steps used in this assay were similar to those described for plasma corticosterone determination (Section 2.7). α1-AGP concentration in plasma was calculated using an equation generated from the slope of the standard curve.

#### 2.9.2. Ceruloplasmin (CPN)

The ceruloplasmin (CPN) concentration was determined by the method of Martinez-Subiela et al. [55]. The method used in this work was based on the rate of coloured product development from CPN and the substrate 1,4-phenylenediamine-dihydrochloride. Approximately, 8.15 g of sodium acetate trihydrate was dissolved in 100 mL distilled water and the pH of the solution was adjusted to 6.2 with glacial acetic acid. Then, 0.246 g of 1,4-phenylenediamine-dihydrochloride (Sigma Chemical, P1519) was added to the prepared buffer and kept in the dark for a minimum of 45 min. A 100 μL of the working solution and 10 μL of samples or standards were loaded into appropriate microplate wells, gently shaken and kept in the dark for 20 minutes. The ceruloplasmin oxidase activity was measured at 550 nm using a microplate reader (Multiskan GO, Thermo Scientific, Waltham, MA, USA). A standard curve was carried out with serial dilution of pig plasma of known CPN concentration, calibrated against a combination of purified CPN (Sigma-Aldrich, St. Louis, MO, USA) and saline buffer to obtain different concentrations of 12.75 (20 μL pig plasma + 60 μL saline buffer), 6.375, 3.187, 1.593, 0.796, 0.398, 0.199, and 0.099 mg/mL CPN. A blank, containing phenylenediamine dihydrochloride solution and distilled water absorbance, was identified and subtracted from the samples and standard absorbance.

### 2.10. Meat Quality

#### 2.10.1. Drip Loss

Drip loss of the meat samples was evaluated using the method described by Honikel [56]. After slaughtering, approximately 25–35 g of fresh meat samples from *pectoralis major* muscle was collected, individually weighed and recorded as the initial weight (W1). Polyethene plastics bags used to pack each sample were sealed and the vacuum packages were stored at 4 °C in the chiller. Then, at 7 days post-storage, the final weight (W2) was measured immediately after removing the samples from the bags and blotted dry. The calculation of the percentage drip loss was done by differences in the final and initial weight of the sample. The sample weight was divided by the initial sample weight after 7 days of storage using the following equation:

(2) $$\mathrm{Drip}\;\mathrm{loss}\;\%=\left(\frac{W1-W2}{W1}\right)\times 100$$

W1 = initial sample weight on day 0; W2 = final sample weight after 7 days of storage.

#### 2.10.2. Cooking Loss

Cooking losses of the meat samples were obtained according to Honikel [56]. Fresh meat samples from *pectoralis major* muscle were weighed individually and recorded as the initial weight (W1). Samples were then cooked in a water bath at 80 °C for 20 min in plastic bags. Following that, the samples were cooled at room temperature and blotted gently and reweighed as a final weight (W2). Cooking loss percentage was quantified as the initial and the final weight difference using the following equation:

(3) $$\mathrm{Cooking}\;\mathrm{loss}\;\%=\left(\frac{W1-W2}{W1}\right)\times 100$$

where W1 = weight before cooking; W2 = weight after cooking. 

#### 2.10.3. Shear Force (Tenderness)

The shear force of the meat sample was determined with the protocol according to Sazili et al. [57]. Upon the determination of cooking loss, the same meat samples proceeded to a shear force determination using a TA.HD plus^®^ texture analyser (Stable Micro Systems, Surrey, UK) having a Volodkevitch bite jaw equipment. Meat samples length were cut in parallel to the direction of the muscle fibres from each sample in triplicate blocks, each of them measuring 1 cm (height) × 1 cm (width) × 2 cm (length). Each block was sheared with the Volodkevitch bite jaw on the middle of the block, perpendicular to the direction of the fibres. Tenderness of meat is inversely comparable to the shear force values. Shear force values were expressed as the average peak positive force value of triplicate blocks from each individual meat sample, and the results were expressed in grams.

#### 2.10.4. Colour

A ColorFlex EZ spectrophotometer (Hunter Associates Laboratory, Inc., Reston, VA, USA) was used for meat colour determination following the International Commission on Illumination (CIE) Lab-values. Before using it, the colourimeter was calibrated against black and white tiles. The frozen meat samples were thawed overnight into a 4 °C chiller. The thawed meat samples were subjected to blooming for 30 min and transferred into the ColorFlex sample cup with the bloomed meat surface facing the base of the cup. Meat colour was measured in triplicate (the cup was rotated clockwise to 90° in the second and third reading) and to obtain the average values of lightness (L*), redness (a*) and yellowness (b*) readings [58].

#### 2.10.5. Meat pH

The breast muscle samples stored at −80 °C were removed and pulverised using a mortar and pestle with presence of liquid nitrogen. Half a gram of each sample was homogenised (Wiggen Hauser^®^ D-500, Berlin, Germany) for 30 seconds in 10 mL ice cold 5 mM sodium iodoacetate (Merck Schuchardt OHG, Hohenbrunn, Germany), and 150 mM KCl solution for 20 seconds to stop glycolysis process (specifically glyceraldehyde 3-phosphate dehydrogenase) and its subsequent lactic acid production [58]. A pre-calibrated pH meter (Mettler-Toledo AG, Zürich, Switzerland) was used for the measurement of pH and calibration was done prior to usage using pH 4.0 and 7.0 buffers.

### 2.11. Statistical Analysis

All statistical analyses were analysed using the 9.4 Version of Statistical Analysis System (SAS) software (SAS Inc., Cary, NC, USA). All data were analysed as 1-way ANOVA using the general linear model procedure. The analysis involved diet as the main effect in a completely randomised design. Where appropriate, means were separated by Duncan’s multiple range test. The statistical significance was considered at *p* < 0.05. 

## 3. Results

### 3.1. Antioxidant Enzyme Activities 

The SOD and GPx activities did not differ (*p* > 0.05) among treatment groups (Table 1). The CAT activity was significantly (*p* < 0.05) enhanced in the RI11 group compared to NC and PC groups. The GSH activity was also significantly (*p* < 0.05) higher in UL4 and RI11 groups compared to NC and PC groups. Birds fed with the RI11 diet had significantly (*p* < 0.05) higher T-AOC activity than their counterparts fed with PC, NC and UL4 diets. The broilers fed with RS5 diet had higher (*p* < 0.05) T-AOC than broilers fed with NC diet. 

### 3.2. Acute Phase Proteins (APPs) and Heat Shock Protein 70 (HSP70)

**Figure 1:**
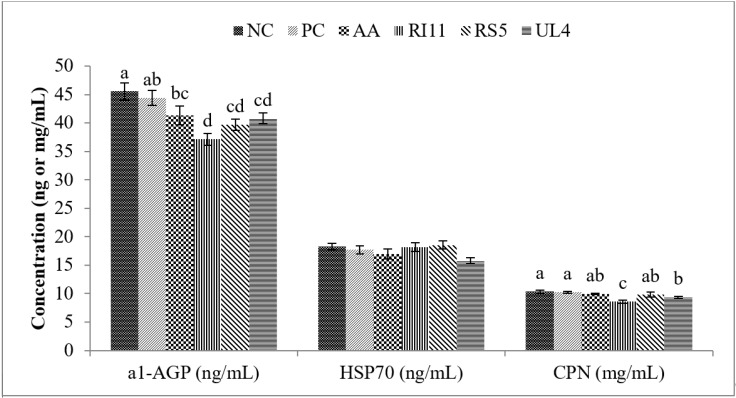
Effect of feeding different postbiotics on plasma acute phase proteins (α1-AGP and CPN), and HSP70 concentrations in heat-stressed broiler chickens. a,b,c,d standard error bars sharing different letters are significantly different (*p* < 0.05). Data are shown as means and standard error (*n* = 12). α1-AGP: alpha-1-acid-glycoprotein, HSP70: heat shock protein 70, CPN: ceruloplasmin. Diets: Negative control (NC): basal diet without any supplementation, positive control (PC): basal diet + 0.02% oxytetracycline (*w*/*w*), AA: basal diet + 0.02% ascorbic acid (*w*/*w*), RI11: basal diet + 0.3% postbiotic RI11 (*v*/*w*), RS5: basal diet + 0.3% postbiotic RS5 (*v*/*w*), UL4: basal diet + 0.3% postbiotic UL4 (*v*/*w*).

The impacts of feeding different postbiotics on APPs (α1-AGP and CPN) and HSP70 in broiler chickens under heat stress conditions are summarised in Figure 1. CPN concentration was significantly (*p* < 0.05) lower in birds fed RI11 diet as compared to the birds fed NC, PC and AA diets. However, difference in CPN was neither observed between the NC and PC groups nor between AA, UL4 and RS5 groups. The RI11 group recorded the lowest α1-AGP concentration compared to other groups. The UL4 group also had significantly lower α1-AGP plasma concentration compared to the NC group. HSP70 concentration was not affected by dietary treatment (*p* > 0.05).

### 3.3. Plasma Corticosterone 

**Figure 2:**
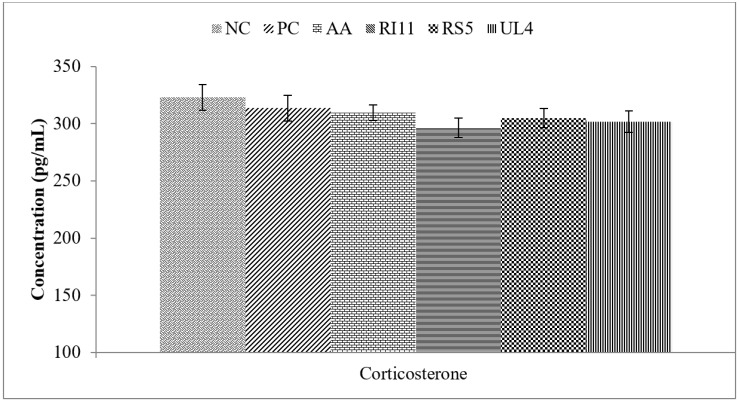
Effect of feeding different postbiotics on plasma corticosterone concentration in heat-stressed broiler chickens. Data represent means and standard error (*n* = 12). Diets: Negative control (NC): basal diet without any supplementation, Positive control (PC): basal diet + 0.02% oxytetracycline (*w*/*w*), AA: basal diet + 0.02% ascorbic acid (*w*/*w*), RI11: basal diet + 0.3% postbiotic RI11 (*v*/*w*), RS5: basal diet + 0.3% postbiotic RS5 (*v*/*w*), UL4: basal diet + 0.3% postbiotic UL4 (*v*/*w*).

The plasma corticosterone level was not affected (*p* > 0.05) by dietary treatment (Figure 2).

### 3.4. Plasma Lipid Profile

Feeding broilers with RI11 diet resulted in lower (*p* < 0.05) total cholesterol as compared with broilers fed NC, PC or AA diets, but not different (*p* > 0.05) when compared with other postbiotic groups (Table 2). No differences (*p* > 0.05) were noted for the cholesterol level between PC, UL4, RS5 and AA groups or between NC and PC treatment groups. Triglyceride and VLDL were lower (*p* < 0.05) in postbiotic groups compared with NC, but not different *(p* > 0.05) from PC and AA treatment groups. The triglyceride and VLDL concentrations were not different (*p* > 0.05) in broilers fed AA, PC and NC diets. The LDL level was lower (*p* < 0.05) in birds fed with the postbiotics and AA diets compared with birds that received the PC and NC diets. No differences (*p* > 0.05) were observed between NC and PC groups for LDL concentration. Dietary treatment had no effect (*p* > 0.05) on HDL concentration.

### 3.5. Meat Quality (pH, Drip Loss, Cooking Loss, Shear Force and Colour) and Lipid Peroxidation (TBARS)

Table 3 shows the effects of different postbiotics supplementation on pH, drip loss, cooking loss, shear force and colour of *pectorals major* muscle from heat-stressed broiler chickens. The pH was significantly higher in the RI11 group as compared with RS5, NC and PC treatment groups, whereas the pH in RS5 group was not different (*p* > 0.05) compared with those of the UL4 and AA groups. The latter treatment groups were higher (*p* < 0.05) in breast meat pH than NC and PC groups. RI11 and UL4 groups had lower (*p* < 0.05) meat drip loss as compared with the meat from the NC treatment group. The drip loss in the meat from heat-stressed broiler chickens was not different between PC, AA, RI11, UL4 and RS5 treatment groups. The cooking losses from RI11 and RS5 postbiotics groups were significantly lower compared to the AA, PC and NC treatment groups. However, there were no significant differences between the latter groups in the cooking loss. Shearing force of *pectoralis major* muscle of heat-stressed broiler chickens was higher (*p* < 0.05) in the NC group compared to AA, RI11, RS5 and UL4 treatment groups. No differences (*p* > 0.05) were recorded for the share force among PC, AA, RI11, UL4 and RS5 treatment groups or between the NC and PC treatment groups. 

Birds fed RI11, UL4 and AA diets had lower (*p* < 0.05) lightness values compared to PC and NC treatment groups. No significant differences were observed for lightness among birds fed on AA, RI11 and UL4 diets or between birds fed RS5, NC and PC diets. The meat yellowness was higher (*p* < 0.05) in the NC, PC and AA treatment groups compared to the RI11 postbiotics group. No differences were observed for redness among all the treatment groups.

For the lipid peroxidation), there were significant reductions (*p* < 0.05) in thigh MDA content of RI11 and UL4 treatment groups compared to PC, NC and AA treatment groups. No differences were observed for MDA in between PC and AA treatment groups. The MDA content of the RS5 group was not different from PC and AA treatment groups.

## 4. Discussion

### 4.1. Antioxidant Activities and Lipid Peroxidation

Heats stress increases core body temperature, which triggers an increment in the production of free radicals leading to oxidative damage [59,60]. Oxidative stress leads to the production of varieties of ROS, including hydroxyl free radical and superoxide anions. Several studies showed that overflow of ROS could damage the biological macromolecules such as proteins and nucleic acids, and produce huge amounts of MDA causing tissue damage, consequently leading to the development of diseases [61]. In birds, the main antioxidant enzymes are glutathione peroxidase (GPx), superoxide dismutase (SOD), catalase (CAT) and glutathione (GSH). These enzymes are a higher order of antioxidant defence acting to transform reactive species into non-radical and non-toxic products [62,63]. 

Postbiotic supplementation in the present study did not affect the SOD and GPx activities. However, the CAT and GSH activities were enhanced in the postbiotic groups, especially in RI11 and UL4 birds as compared with the negative control and other treatment groups. Likewise, two studies reported that broilers under heat challenge had increased activities of CAT, GPx, GSH and SOD [60,64]. Heat-stressed birds fed with postbiotics also had significantly higher T-AOC in the present study. The present study results were in line with the findings of Wang et al. [65] who found that broilers fed probiotic *Lactobacillus johnsonii* BS15 had significantly higher blood T-AOC, SOD and CAT activities compared to a negative control group. Hence, the dietary postbiotics showed the capacity to improve antioxidant activities (concentrations of CAT, GSH and T-AOC) in the plasma of heat-stressed broilers. Postbiotics are a natural source of antimicrobial and antioxidant that can safely alleviate the stress and improve the health of animals. This finding is consistent with Akbarian et al. [66], who observed dietary supplementation with another natural source of antioxidant (*Xanthorrhiza* and *Origanum compactum*) in heat-stressed broilers, which led to increased mRNA levels of SOD, GPx, and CAT, in the hepatic system. As postbiotics possess probiotic characteristics [34,39,40,43,67,68], probiotic studies can provide useful information to understand how postbiotics could improve the antioxidant capability and develop the oxidative resistance in the body under heat stress. Several studies reported that the supplementation of probiotics in poultry diets reduced the adverse effects of oxidative stress and enhanced the activity of antioxidant enzymes [61,69], which might reduce cell damage by inhibiting the production of ROS and finally improving the health of animals [70,71]. Our results were consistent with Shen et al. [72] who reported that blood antioxidant capacities were significantly intensified by the incorporation of probiotic *L. plantarum* in the diets and promoted growth performance in broilers.

This study is the first attempt to provide data on the effect of postbiotics on antioxidant activities in heat-stressed broilers. However, probiotics have been reported for their ROS removal capacity and promoting broiler health under normal [61] and high-temperature conditions [73]. 

Generally, broiler chickens are characterised by their high lipid contents which increase the exposure to lipid peroxidation, which could harm the body due to ROS production to maintain nutritional and physiological demands. Lipid peroxidation is a sequela of reduced antioxidant protection as the production of free radicals and ROS increases. The MDA level is often used as an endogenous reflection of lipid peroxidation [74]. 

Antioxidant enzymes such as CAT, GPx, GSH and SOD are vital in scavenging excess ROS at the cellular level, thereby affecting the stability of lipid peroxidation of broiler meat during storage [75]. In this study, there was a significant reduction in MDA content in broilers fed RI11 and UL4 under heat stress compared to negative control and other groups. The postbiotics herein showed probiotic antioxidant benefits and could promote the function of CAT and other antioxidant enzymes. This may be associated to the postbiotic ability to confer effective antioxidant protection in reducing lipid peroxidation during the growth phase of the birds, despite being under heat challenge. 

### 4.2. Acute Phase Proteins, HSP70 and Corticosterone 

APPs are blood proteins majorly produced in the hepatic system and their concentrations change under conditions such as inflammatory reactions, infections, and tissue injuries [4]. APPs also function to restore homeostasis and prevent microbial proliferation following stimulation by non-specific innate immune cells [76]. In this study, the CPN and α1-AGP concentrations were significantly reduced in the heat-stressed birds fed either with postbiotics (RI11, RS5, UL4) or ascorbic acid (AA) compared to the negative control. These findings indicate the beneficial role of the postbiotics (especially RI11) in improving immune response so as to annul the negative effect of heat stress on the broiler chickens. The capacity of ascorbic acid to produce such a beneficial effect was also illustrated, though the concentrations of the APPs were still higher compared with the RI11 fed birds. 

Various authors have reported different response kinetics of APPs in avian species. APPs such as AGP were shown to be faster reacting than CPN upon exogenous administration of corticosterone [77]. In contrast, corticosterone was markedly elevated after 2 h of feed deprivation compared to AGP and CPN which showed similar response only after 30 h [4]. Such discrepancies suggest species differences in either stress exposure or capacity for APPs synthesis. 

In this study, despite the lower corticosterone level in broilers fed with postbiotics as compared to the NC, the effect was not significant. Changes in corticosterone levels could be influenced by the duration of heat exposure, and the benefits of postbiotic supplementation might be masked during chronic heat stress. As reported by Sohail et al. [78], broilers supplemented with prebiotic and probiotic mixture recorded lower serum corticosterone concentrations on day 21 following heat exposure compared with the negative control group.

Another important plasma protein is HSP70, as their levels increase during cellular insult, which is useful in predicting the degree of thermal stress in broilers [77]. HSP70 is a type of heat shock protein that is highly conserved and promptly synthesised in response to stressors such as feed restriction, crating, transportation, unpleasant human contact, and elevated temperature [77,79,80,81]. 

In this study, the HSP70 level was not significantly different between the heat-stressed broilers supplemented with postbiotics, antibiotics, ascorbic acid, and the negative control. HSP70 acts as a chaperone through interaction between proteins to defend synthesised proteins against additional injury [82]. Hence, HSP70 induction guards against stresses such as hyperthermia, ischemia, and inflammation. For instance, HSP70 levels increased significantly in broilers exposed to heat stress [4,80]. Broilers restricted from feeding had significantly lower HSP70 and *S. enteritidis* colonization compared with the control group after heat exposure [83]. Ascorbic acid also induced increased body temperature in heat-stressed chickens, which correlated with the HSP70 response [1]. Another study reported that the concentration of HSP70 decreased after exposure the birds to long-term heat stress and this could be that the birds developed tolerance to heat after long heat stress, or due to material insufficiency after long-term stressing [84,85]. These results are inconsistent with the present study, as we observed no difference in HSP70 response following postbiotic and ascorbic acid supplementation. However, the expression of HSP70 could be influenced by various factors including the type of target organ and age of the birds [86]. Yan et al. [87] showed that HSP70 induction is organ dependent following the induction of the protein in heat-stressed chickens. Differences in the management system, breeds, and the degree of thermal stress could explain the inconsistent result between the present study and other related studies.

### 4.3. Plasma Lipid Profile

The birds supplemented with various types of postbiotics had lower total cholesterol and triglyceride levels. Nevertheless, the effect of total cholesterol in the former groups was not different compared with AA fed birds. The present result is in agreement with that of [25] who found decreased cholesterol levels following supplementation with various combinations of postbiotics. Kareem [88] reported that supplementation of postbiotics and inulin in broiler diets decreased serum cholesterol, triglyceride and LDL compared with the antibiotic and negative control group. Similar results were found in the reduction of cholesterol profile in plasma of laying hens [34], post-weaning rats [50] and piglets [89] fed postbiotics produced from *L. plantarum*.

Our result corroborated that of [35], where plasma cholesterol concentration was reduced in egg yolk following dietary feeding with postbiotics. *L. plantarum* metabolites have been reported to have a cholesterol-lowering effect in rats [49]. This could be related to the increase in the population of LAB, as found in this study. Through conjugation, the primary bile acids are transformed into secondary products by the intrinsic ability of LAB. Therefore, the volume of bile acid in the intestine is reduced. Another likely pathway for the effect observed in this study is the enhancement of both faecal excretion and turnover of bile acids [49]. 

Cholesterol is a precursor for bile acid synthesis, which explains the reason for the higher rate of cholesterol break down [90]. Postbiotics used in this study could increase the population of lactic acid bacteria, production of enzymes disintegrating bile salts and de-conjugating them in the gut, as well as reduction of the gut pH that can be efficient in decreasing the cholesterol of blood by reducing non-conjugate bile acids solvability at low pH, leading to less absorption from the intestine and more excretion in the faeces [88]. Other authors have demonstrated that cholesterol levels can be reduced when cholesterol is incorporated into the bacterial cellular membrane [91]. In the present study, postbiotics, especially RI11 supplemented in broiler diets under heat stress, showed the same trend of previous studies in terms of reducing plasma cholesterol profile levels.

### 4.4. Meat Quality 

Aside from the health implications, the quality of the meat obtained from the chicken muscle is crucial in the economics of meat processing industries. The water holding capacity (WHC) of meat is indicated by the drip and cooking losses, while the latter determines profit in meat sales [92]. 

In this study, the higher drip losses in the meat of heat-stressed broilers fed only a basal diet are comparable to earlier works reporting the adverse effect of exposure of broilers to heat stress on meat quality. Both acute and chronic heat stress exposure could negatively affect meat quality [93,94]. The former occurs by alteration in aerobic metabolism, glycolysis, and intramuscular deposition of fat, thus leading to pale meat colour, reduced WHC, and higher shear force [95,96]. Acute heat stress induces changes in blood acid-base status and integrity of muscle membrane [97]. Findings from the present study showed broilers fed with postbiotics had significantly lower drip loss and cooking loss than the NC, OTC and AA fed groups. This is consistent with other studies conducted using postbiotics and probiotics. Although the experiments were conducted under normal ambient temperature, broilers fed combinations of postbiotics and inulin had lower drip loss as compared with control groups [31]. Additionally, dietary probiotics resulted in significant reductions in drip loss in the breast muscle of chickens [98]. 

Ali [99] also observed no significant change in shear force in breast muscle of broilers fed with or without probiotics. Shearing force in the present study was higher (*p* < 0.05) in the antibiotic and NC birds as compared with those fed various postbiotics. This might be due to the capacity of the postbiotics to annul the effect of thermal stress in the broilers as it relates to antioxidant enzyme activities. Similar findings were reported in one study based on the lower shear force in the birds fed probiotics [98]. Another study reported that the meat pH, colour, drip loss, cooking loss and tenderness were improved by feeding probiotics which induced the differential expression of carbohydrate metabolism, cytoskeleton, chaperone and transportation proteins. These proteins participate in carbohydrate and energy metabolism as well as tight junction pathways, suggesting roles in the organization of meat quality improvement [100]. The same previous study documented that the improvement in the meat quality traits in the broilers fed probiotic *Enterococcus faecium* may be attributed to the up-regulation of the protein’s related substrate metabolism, antioxidant and immune systems which lead to alterations of the pentose phosphate and citric acid pathways and increase the metabolism of amino acids and improve antioxidant and immune capacity leading to improved muscle biochemical indexes (pH, colour, WHC and texture). The postbiotic inclusion in the broiler diets under heat stress is expected to deliver the same action of probiotics on the meat quality, as mentioned.

Another indicator of meat quality is its pH [101]. The pH value of 6.0 is considered good for broiler breast meat [101]. The pH of the breast meat of all the treatment groups ranged from 5.7–6.0, thus signifying good quality. The pH of the birds fed with postbiotics (RI11, RS5, UL4) was higher (*p* < 0.05) compared with the PC and NC groups. This is another indication of the role of the postbiotics administered to the heat-stressed birds in maintaining the meat quality. Our observation is different in comparison to the study where dietary supplementation with postbiotics and inulin resulted in significantly lower pH in broiler meat [31]. The fact that the birds in this study were under heat challenge could explain the disparity between our results and the previous study. Nevertheless, since the final pH of the breast meat was within the normal range, the difference offers no significant impact. 

Meat colour as an indicator of broiler meat quality was also examined in the present study. Broilers fed postbiotics (UL4, RI11 and RS5) and ascorbic acid (AA) had significantly lower lightness values compared to the negative control and antibiotic group, while birds fed RI11 had the lowest lightness level. Lightness is used as a scale of breast muscle colour [102]. Myoglobin in the meat is responsible for absorbing green light, leading to the more yellowish and less reddish appearance of meat [103]. Therefore, the lower lightness in the treated broiler chickens could be attributed to the higher pH of the muscle [104]. Such colour changes were reported in previous studies following probiotics feeding [99,104,105]. Additionally, the redness level was not significantly different between the treatment groups in the present study, which is consistent with the results of [106]. However, [31] reported changes in redness level in breast meat after combined feeding of postbiotics and inulin. 

Based on the review by [97], we suggest there are three main pathways through which postbiotics exert their action in annulling the adverse effects of heat stress on meat quality in broiler chickens. First, is the prevention of a rapid drop in pH which could be detrimental to meat quality. Low pH has been associated with low redness, high drip and cooking losses in breast meat of chickens [107,108,109]. Second, the enhancement of antioxidant activities of GPx, SOD, and catalase, is vital to alleviate the effect of heat stress on meat quality through oxidative damages. The third likely mechanism is lowering the corticoid hormone levels (secretion of corticosterone). Sato et al. [110] reported that corticosterone accelerates ROS production, this could result in pale and high drip loss of broiler meat. 

## 5. Conclusions

The findings of the current study presented that feeding postbiotics, especially RI11, enhanced the antioxidant activities, meat quality (pH, WHC, colour and tenderness) and reduced acute phase proteins (AGP and CPN), plasma cholesterol and lipid peroxidation in broiler chickens exposed to heat stress conditions. Hence, postbiotics can be used as antioxidant agents in production of poultry under hot weather conditions.

## Figures and Tables

**Table 1:** Antioxidant enzymes activities in heat-stressed broilers fed with three different postbiotics.

Parameter	Diets ^1^						SEM	*p*-Values
	NC	PC	AA	RI11	RS5	UL4		
T-AOC (mM)	0.63 ^c^	0.67 ^b,c^	0.68 ^b,c^	0.79 ^a^	0.76 ^a,b^	0.69 ^b,c^	0.017	0.028
GPx (µmol/L)	529.65	569.23	530.2	678.22	569.01	672.9	24.64	0.286
CAT (U/L)	3.95 ^b^	4.27 ^b^	4.46 ^a,b^	5.01 ^a^	4.39 ^a,b^	4.48 ^a,b^	0.096	0.046
GSH (µM)	42.29 ^b^	42.20 ^b^	45.30 ^a,b^	46.72 ^a^	45.71 ^a,b^	46.22 ^a^	0.534	0.026
SOD (U/mL)	0.955	0.959	0.981	1.190	0.944	1.069	0.028	0.129

^a,b,c^ Means within a row sharing different superscript letters are significantly different (*p* < 0.05). ^1^ Diets: NC (negative control): basal diet without any supplementation, PC (positive control): basal diet + 0.02% oxytetracycline (*w*/*w*), AA: basal diet + 0.02% ascorbic acid (*w*/*w*), RI11: basal diet + 0.3% postbiotic RI11 (*v*/*w*), RS5: basal diet + 0.3% postbiotic RS5 (*v*/*w*), UL4: basal diet + 0.3% postbiotic UL4 (*v*/*w*). SEM: Standard error of means. T-AOC: Total-antioxidant capacity; GPx: Glutathione peroxidase; CAT: Catalase; GSH: Glutathione; SOD: Superoxide dismutase.

**Table 2:** Plasma lipid profiles (mmol/L) in heat-stressed broiler chickens fed with three different postbiotics.

Parameter	Diets ^1^						SEM	*p*-Values
	NC	PC	AA	RI11	RS5	UL4		
Cholesterol	3.46 ^a^	2.816 ^a,b^	2.46 ^b^	1.78 ^c^	1.916 ^b,c^	2.033 ^b,c^	0.149	0.003
Triglyceride	1.016 ^a^	0.77 ^a,b^	0.64 ^a,b^	0.416 ^b^	0.456 ^b^	0.34 ^b^	0.070	0.043
VLDL	0.203 ^a^	0.154 ^a,b^	0.128 ^a,b^	0.083 ^b^	0.091 ^b^	0.068 ^b^	0.014	0.043
LDL	0.783 ^a^	0.638 ^a^	0.446 ^b^	0.403 ^b^	0.386 ^b^	0.408 ^b^	0.033	<0.0001
HDL	1.1883	1.44	1.415	2.19	1.8783	1.74	0.104	0.126

^a,b,c^ Means within a row sharing different superscript letters are significantly different (*p* < 0.05). ^1^ Diets: NC (negative control): basal diet without any supplementation, PC (positive control): basal diet + 0.02% oxytetracycline (*w*/*w*), AA: basal diet + 0.02% ascorbic acid (*w*/*w*), RI11: basal diet + 0.3% postbiotic RI11 (*v*/*w*), RS5: basal diet + 0.3% postbiotic RS5 (*v*/*w*), UL4: basal diet + 0.3% postbiotic UL4 (*v*/*w*). SEM: Standard error of means. VLDL: very low density lipoprotein, LDL: low density lipoprotein, HDL: high density lipoprotein.

**Table 3:** Breast meat quality and thigh lipid peroxidation in heat-stressed broilers fed with three different postbiotics.

Parameter	Diets ^1^						SEM	*p*-Values
	NC	PC	AA	RI11	RS5	UL4		
pH	5.72 ^c^	5.77 ^c^	0.0007	6.01 ^a^	5.85 ^b,c^	5.95 ^a,b^	0.02	0.0007
Drip loss %	3.90 ^a^	2.90 ^a,b^	0.024	2.32 ^b^	2.87 ^a,b^	2.20 ^b^	0.15	0.024
Cooking loss %	21.64 ^a,b^	23.13 ^a^	0.009	17.89 ^c^	19.20 ^c^	19.38 ^b,c^	0.39	0.009
Shear force (g)	1113.8 ^a^	1021.6 ^a,b^	0.042	922.6 ^b^	949.1 ^b^	965.8 ^b^	18.25	0.042
Colour								
L* (Lightness)	49.02 ^a^	48.47 ^a^	45.82 ^b,c^	44.84 ^c^	47.32 ^ab^	45.96 ^b,c^	0.34	0.001
a* (Redness)	4.51	4.58	4.81	5.27	5.01	4.92	0.18	0.877
b* (Yellowness)	14.54 ^a,b^	15.60 ^a^	15.41 ^a^	12.63 ^c^	14.10 ^a,b,c^	13.18 ^b,c^	0.23	0.0003
TBARS (µg MDA/g thigh meat)	9.85 ^a^	8.91 ^a,b^	9.04 ^a,b^	7.01 ^c^	8.22 ^b,c^	7.37 ^c^	0.213	<0.0001

^a,b,c^ Means within a row sharing different superscript letters are significantly different (*p* < 0.05). ^1^ Diets: NC (negative control): basal diet without any supplementation, PC (positive control): basal diet + 0.02% oxytetracycline (*w*/*w*), AA: basal diet + 0.02% ascorbic acid (*w*/*w*), RI11: basal diet + 0.3% postbiotic RI11 (*v*/*w*), RS5: basal diet + 0.3% postbiotic RS5 (*v*/*w*), UL4: basal diet + 0.3% postbiotic UL4 (*v*/*w*). SEM: Standard error of means, L*: Lightness, a*: Redness, b*: Yellowness, TBARS: Thiobarbituric acid reactive substances, MDA: Mainlymalondialdehyde.

## References

[1] Mahmoud K.Z., Edens F.W., Eisen E.J., Havenstein G.B. (2003). Effect of ascorbic acid and acute heat exposure on heat shock protein 70 expression by young white Leghorn chickens. Comp. Biochem. Physiol. C Toxicol. Pharmacol..

[2] Mahmoud K.Z., Edens F.W., Eisen E.J., Havenstein G.B. (2004). Ascorbic acid decreases heat shock protein 70 and plasma corticosterone response in broilers (*Gallus gallus domesticus*) subjected to cyclic heat stress. Comp. Biochem. Physiol. Part B Biochem. Mol. Biol..

[3] He X., Lu Z., Ma B., Zhang L., Li J., Jiang Y., Zhou G., Gao F. (2018). Effects of chronic heat exposure on growth performance, intestinal epithelial histology, appetite-related hormones and genes expression in broilers. J. Sci. Food Agric..

[4] Najafi P., Zulkifli I., Jajuli N.A., Farjam A.S., Ramiah S.K., Amir A.A., O‘Reily E., Eckersall D. (2015). Environmental temperature and stocking density effects on acute phase proteins, heat shock protein 70, circulating corticosterone and performance in broiler chickens. Int. J. Biometeorol..

[5] Lykkesfeldt J., Svendsen O. (2007). Oxidants and antioxidants in disease: Oxidative stress in farm animals. Vet. J..

[6] Matés J.M., Pérez-Gómez C., De Castro I.N. (1999). Antioxidant enzymes and human diseases. Clin. Biochem..

[7] Mager W.H., De Kruijff A. (1995). Stress-induced transcriptional activation. Microbiol. Mol. Biol. Rev..

[8] Iwagami Y. (1996). Changes in the ultrastructure of human cells related to certain biological responses under hyperthermic culture conditions. Hum. Cell.

[9] Halliwell B., Cross C.E. (1994). Oxygen-derived species: Their relation to human disease and environmental stress. Environ. Health Perspect..

[10] Halliwell B. (2001). Role of free radicals in the neurodegenerative diseases. Drugs Aging.

[11] Rahimi S., Khaksefidi A. (2006). A comparison between the effects of a probiotic (Bioplus 2B) and an antibiotic (virginiamycin) on the performance of broiler chickens under heat stress condition. Iran. J. Vet. Res..

[12] Zulkifli I., Abdullah N., Azrin N.M., Ho Y. (2000). Growth performance and immune response of two commercial broiler strains fed diets containing *Lactobacillus* cultures and oxytetracycline under heat stress conditions. Br. Poult. Sci..

[13] Ramiah S.K., Zulkifli I., Rahim N.A.A., Ebrahimi M., Meng G.Y. (2014). Effects of two herbal extracts and virginiamycin supplementation on growth performance, intestinal microflora population and fatty acid composition in broiler chickens. Asian-Australas. J. Anim. Sci..

[14] Odore R., De Marco M., Gasco L., Rotolo L., Meucci V., Palatucci A., Rubino V., Ruggiero G., Canello S., Guidetti G. (2015). Cytotoxic effects of oxytetracycline residues in the bones of broiler chickens following therapeutic oral administration of a water formulation. Poult. Sci..

[15] Shazali N., Foo H.L., Loh T.C., Choe D.W., Rahim R.A. (2014). Prevalence of antibiotic resistance in lactic acid bacteria isolated from the faeces of broiler chicken in Malaysia. Gut Pathog..

[16] Regulation E. (2003). No 1831/2003 of the European Parliament and Council of 22 September 2003 on additives for use in animal nutrition. Off. J. Eur. Commun..

[17] Van Boeckel T.P., Brower C., Gilbert M., Grenfell B.T., Levin S.A., Robinson T.P., Teillant A., Laxminarayan R. (2015). Global trends in antimicrobial use in food animals. Proc. Natl. Acad. Sci. USA.

[18] Tajkarimi M., Ibrahim S.A. (2011). Antimicrobial activity of ascorbic acid alone or in combination with lactic acid on *Escherichia coli* O157: H7 in laboratory medium and carrot juice. Food Control.

[19] Verghese R.J., Ramya S., Kanungo R. (2017). In vitro Antibacterial Activity of Vitamin C and in Combination with Ciprofloxacin against Uropathogenic *Escherichia coli*. J. Clin. Diagn. Res..

[20] Njoku P. (1986). Effect of dietary ascorbic acid (vitamin C) supplementation on the performance of broiler chickens in a tropical environment. Anim. Feed Sci. Technol..

[21] Kadim I., Al-Qamshui B., Mahgoub O., Al-Marzooqi W., Johnson E. (2008). Effect of seasonal temperatures and ascorbic acid supplementation on performance of broiler chickens maintained in closed and open-sided houses. Int. J. Poult. Sci..

[22] Kutlu H., Forbes J. (1993). Changes in growth and blood parameters in heat-stressed broiler chicks in response to dietary ascorbic acid. Livest. Prod. Sci..

[23] Sahin K., Sahin N., Kucuk O. (2003). Effects of chromium, and ascorbic acid supplementation on growth, carcass traits, serum metabolites, and antioxidant status of broiler chickens reared at a high ambient temperature (32 °C). Nutr. Res..

[24] Ferreira I., Matos Junior J., Sgavioli S., Vicentini T., Morita V., Boleli I. (2015). Vitamin C prevents the effects of high rearing temperatures on the quality of broiler thigh meat. Poult. Sci..

[25] Thanh N.T., Loh T.C., Foo H.L., Hair-Bejo M., Azhar B.K. (2009). Effects of feeding metabolite combinations produced by *Lactobacillus plantarum* on growth performance, faecal microbial population, small intestine villus height and faecal volatile fatty acids in broilers. Br. Poult. Sci..

[26] Aguilar-Toalá J., Garcia-Varela R., Garcia H., Mata-Haro V., González-Córdova A., Vallejo-Cordoba B., Hernández-Mendoza A. (2018). Postbiotics: An evolving term within the functional foods field. Trends Food Sci. Technol..

[27] Kareem K.Y., Ling F.H., Chwen L.T., Foong O.M., Asmara S.A. (2014). Inhibitory activity of postbiotic produced by strains of *Lactobacillus plantarum* using reconstituted media supplemented with inulin. Gut Pathog..

[28] Choe D.W., Foo H.L., Loh T.C., Hair-Bejo M., Awis Q.S. (2013). Inhibitory property of metabolite combinations produced from *Lactobacillus plantarum* strains. Pertanika J. Trop. Agric. Sci..

[29] Thanh N.T., Chwen L.T., Foo H.L., Hair-Bejo M., Kasim A.B. (2010). Inhibitory activity of metabolites produced by strains of *Lactobacillus plantarum* isolated from Malaysian fermented food. Int. J. Probiot. Prebiot..

[30] Van Thu T., Foo H.L., Loh T.C., Bejo M.H. (2011). Inhibitory activity and organic acid concentrations of metabolite combinations produced by various strains of *Lactobacillus plantarum*. Afr. J. Biotechnol..

[31] Kareem K.Y., Loh T.C., Foo H.L., Akit H., Samsudin A.A. (2016). Effects of dietary postbiotic and inulin on growth performance, IGF1 and GHR mRNA expression, faecal microbiota and volatile fatty acids in broilers. BMC Vet. Res..

[32] Loh T.C., Thanh N.T., Foo H.L., Hair-Bejo M., Azhar B.K. (2010). Feeding of different levels of metabolite combinations produced by *Lactobacillus plantarum* on growth performance, fecal microflora, volatile fatty acids and villi height in broilers. Anim. Sci. J..

[33] Rosyidah M., Loh T., Foo H., Cheng X., Bejo M. (2011). Effect of feeding metabolites and acidifier on growth performance, faecal characteristics and microflora in broiler chickens. J. Anim. Vet. Adv..

[34] Loh T.C., Choe D.W., Foo H.L., Sazili A.Q., Bejo M.H. (2014). Effects of feeding different postbiotic metabolite combinations produced by *Lactobacillus plantarum* strains on egg quality and production performance, faecal parameters and plasma cholesterol in laying hens. BMC Vet. Res..

[35] Choe D.W., Loh T.C., Foo H.L., Hair-Bejo M., Awis Q.S. (2012). Egg production, faecal pH and microbial population, small intestine morphology, and plasma and yolk cholesterol in laying hens given liquid metabolites produced by *Lactobacillus plantarum* strains. Br. Poult. Sci..

[36] Loh T.C., Thu T.V., Foo H.L., Bejo M.H. (2013). Effects of different levels of metabolite combination produced by *Lactobacillus plantarum* on growth performance, diarrhoea, gut environment and digestibility of postweaning piglets. J. Appl. Anim. Res..

[37] Thu T.V., Loh T.C., Foo H.L., Yaakub H., Bejo M.H. (2011). Effects of liquid metabolite combinations produced by *Lactobacillus plantarum* on growth performance, faeces characteristics, intestinal morphology and diarrhoea incidence in postweaning piglets. Trop. Anim. Health Prod..

[38] Izuddin W.I., Loh T.C., Samsudin A.A., Foo H.L. (2018). In vitro study of postbiotics from *Lactobacillus plantarum* RG14 on rumen fermentation and microbial population. Rev. Bras. Zootec..

[39] Izuddin W.I., Loh T.C., Foo H.L., Samsudin A.A., Humam A.M. (2019). Postbiotic *L. plantarum* RG14 improves ruminal epithelium growth, immune status and upregulates the intestinal barrier function in post-weaning lambs. Sci. Rep..

[40] Kareem K.Y., Loh T.C., Foo H.L., Asmara S.A., Akit H. (2016). Influence of postbiotic RG14 and inulin combination on cecal microbiota, organic acid concentration, and cytokine expression in broiler chickens. Poult. Sci..

[41] Kareem K.Y., Loh T.C., Foo H.L., Asmara S.A., Akit H., Abdulla N.R., Ooi M.F. (2015). Carcass, meat and bone quality of broiler chickens fed with postbiotic and prebiotic combinations. Int. J. Probiot. Prebiot..

[42] Loh T., Thanh N., Foo H., Hair-Bejo M. (2013). Effects of feeding metabolite combinations from *Lactobacillus plantarum* on plasma and breast meat lipids in Broiler Chickens. Rev. Bras. Cienc. Avic..

[43] Humam A.M., Loh T.C., Foo H.L., Samsudin A.A., Mustapha N.M., Zulkifli I., Izuddin W.I. (2019). Effects of Feeding Different Postbiotics Produced by *Lactobacillus plantarum* on Growth Performance, Carcass Yield, Intestinal Morphology, Gut Microbiota Composition, Immune Status, and Growth Gene Expression in Broilers under Heat Stress. Animals.

[44] Izuddin W.I., Humam A.M., Loh T.C., Foo H.L., Samsudin A.A. (2020). Dietary Postbiotic *Lactobacillus plantarum* Improves Serum and Ruminal Antioxidant Activity and Upregulates Hepatic Antioxidant Enzymes and Ruminal Barrier Function in Post-Weaning Lambs. Antioxidants.

[45] He Z., Wang X., Li G., Zhao Y., Zhang J., Niu C., Zhang L., Zhang X., Ying D., Li S. (2015). Antioxidant activity of prebiotic ginseng polysaccharides combined with potential probiotic *Lactobacillus plantarum* C88. Int. J. Food Sci. Technol..

[46] Ji K., Jang N.Y., Kim Y.T. (2015). Isolation of Lactic Acid Bacteria Showing Antioxidative and Probiotic Activities from Kimchi and Infant Feces. J. Microbiol. Biotechnol..

[47] Moghadam M.S., Foo H.L., Leow T.C., Rahim R.A., Loh T.C. (2010). Novel Bacteriocinogenic *Lactobacillus plantarum* Strains and Their Differentiation by Sequence Analysis of 16 S rDNA, 16 S-23 S and 23 S-5 S Intergenic Spacer Regions and Randomly Amplified Polymorphic DNA Analysis. Food Technol. Biotechnol..

[48] Lim Y.H., Foo H.L., Loh T.C., Mohamad R., Abdullah N. (2019). Comparative studies of versatile extracellular proteolytic activities of lactic acid bacteria and their potential for extracellular amino acid productions as feed supplements. J. Anim. Sci. Biotechnol..

[49] Foo H., Loh T., Law F., Lim Y., Kuflin C., Rusul G. (2003). Effect of feeding *L. plantarum* I-UL4 isolated from Malaysian Tempeh on growth performance, fecla flora and lactic acid bacteria and plasma cholesterol concentrations in post weaning rats. J. Food Sci. Biotechnol..

[50] Loh T., Chong S., Foo H., Law F. (2009). Effects on growth performance, faecal microflora and plasma cholesterol after supplementation of spray-dried metabolite to postweaning rats. Czech J. Anim. Sci..

[51] Malaysian Standard M. (2009). Halal Food—Production, Preparation, Handling and Storage—General Guideline.

[52] DeLong D.M., DeLong E.R., Wood P.D., Lippel K., Rifkind B.M. (1986). A comparison of methods for the estimation of plasma low-and very low-density lipoprotein cholesterol: The Lipid Research Clinics Prevalence Study. JAMA.

[53] Lynch S.M., Frei B. (1993). Mechanisms of copper-and iron-dependent oxidative modification of human low density lipoprotein. J. Lipid Res..

[54] Mercier Y., Gatellier P., Viau M., Remignon H., Renerre M. (1998). Effect of dietary fat and vitamin E on colour stability and on lipid and protein oxidation in turkey meat during storage. Meat Sci..

[55] Martinez-Subiela S., Tecles F., Ceron J. (2007). Comparison of two automated spectrophotometric methods for ceruloplasmin measurement in pigs. Res. Vet. Sci..

[56] Honikel K.O. (1998). Reference methods for the assessment of physical characteristics of meat. Meat Sci..

[57] Sazili A.Q., Parr T., Sensky P.L., Jones S.W., Bardsley R.G., Buttery P.J. (2005). The relationship between slow and fast myosin heavy chain content, calpastatin and meat tenderness in different ovine skeletal muscles. Meat Sci..

[58] American Meat Science Association (2012). AMSA Meat Color Measurement Guidelines.

[59] Akbarian A., Michiels J., Degroote J., Majdeddin M., Golian A., De Smet S. (2016). Association between heat stress and oxidative stress in poultry; mitochondrial dysfunction and dietary interventions with phytochemicals. J. Anim. Sci. Biotechnol..

[60] Altan O., Pabuccuoglu A., Altan A., Konyalioglu S., Bayraktar H. (2003). Effect of heat stress on oxidative stress, lipid peroxidation and some stress parameters in broilers. Br. Poult. Sci..

[61] Bai K., Huang Q., Zhang J., He J., Zhang L., Wang T. (2017). Supplemental effects of probiotic *Bacillus subtilis* fmbJ on growth performance, antioxidant capacity, and meat quality of broiler chickens. Poult. Sci..

[62] Surai P.F. (2015). Silymarin as a Natural Antioxidant: An Overview of the Current Evidence and Perspectives. Antioxidants.

[63] Ko Y., Yang H., Jang I. (2004). Effect of conjugated linoleic acid on intestinal and hepatic antioxidant enzyme activity and lipid peroxidation in broiler chickens. Asian-Australas. J. Anim. Sci..

[64] Yang P.C., Tu Y.H., Perdue M.H., Oluwole C., Struiksma S. (2009). Regulatory effect of heat shock protein 70 in stress-induced rat intestinal epithelial barrier dysfunction. N. Am. J. Med. Sci..

[65] Wang H., Ni X., Qing X., Liu L., Xin J., Luo M., Khalique A., Dan Y., Pan K., Jing B. (2018). Probiotic *Lactobacillus johnsonii* BS15 improves blood parameters related to immunity in broilers experimentally infected with subclinical necrotic enteritis. Front. Microbiol..

[66] Akbarian A., Michiels J., Golian A., De Smet S. (2014). Fourteen days cyclic heat challenge and feeding oreganum compactum and curcuma xanthorrhiza essential oils: Effects on antioxidant system of broilers. Commun. Agric. Appl. Biol. Sci..

[67] Foo H.L., Loh T.C., Abdul Mutalib N.E., Rahim R.A., Faintuch J., Faintuch S. (2019). The myth and therapeutic potentials of postbiotics. Microbiome and Metabolome in Diagnosis, Therapy, and Other Strategic Applications: Academic Press.

[68] Gao J., Li Y., Wan Y., Hu T., Liu L., Yang S., Gong Z., Zeng Q., Wei Y., Yang W. (2019). A novel postbiotic from *Lactobacillus rhamnosus* GG with a beneficial effect on intestinal barrier function. Front. Microbiol..

[69] Deng W., Dong X., Tong J., Zhang Q. (2012). The probiotic *Bacillus licheniformis* ameliorates heat stress-induced impairment of egg production, gut morphology, and intestinal mucosal immunity in laying hens. Poult. Sci..

[70] Havenaar R., Ten Brink B., Huis Veld J.H.J., Fuller R. (1992). Selection of strains for probiotic use. Probiotics: The Scientific Basis.

[71] Li N., Yu H., Liu H., Wang Y., Zhou J., Ma X., Wang Z., Sun C., Qiao S. (2019). Horizontal transfer of vanA between probiotic *Enterococcus faecium* and *Enterococcus faecalis* in fermented soybean meal and in digestive tract of growing pigs. J. Anim. Sci. Biotechnol..

[72] Shen X., Yi D., Ni X., Zeng D., Jing B., Lei M., Bian Z., Zeng Y., Li T., Xin J. (2014). Effects of *Lactobacillus plantarum* on production performance, immune characteristics, antioxidant status, and intestinal microflora of bursin-immunized broilers. Can. J. Microbiol..

[73] Cramer T.A., Kim H.W., Chao Y., Wang W., Cheng H.W., Kim Y.H.B. (2018). Effects of probiotic (*Bacillus subtilis*) supplementation on meat quality characteristics of breast muscle from broilers exposed to chronic heat stress. Poult. Sci..

[74] Yang R.L., Li W., Shi Y.H., Le G.W. (2008). Lipoic acid prevents high-fat diet-induced dyslipidemia and oxidative stress: A microarray analysis. Nutrition.

[75] Bai W.K., Zhang F.J., He T.J., Su P.W., Ying X.Z., Zhang L.L., Wang T. (2016). Dietary Probiotic *Bacillus subtilis* Strain fmbj Increases Antioxidant Capacity and Oxidative Stability of Chicken Breast Meat during Storage. PLoS ONE.

[76] Najafi P., Zulkifli I., Soleimani A.F., Goh Y.M. (2016). Acute phase proteins response to feed deprivation in broiler chickens. Poult. Sci..

[77] Zulkifli I., Najafi P., Nurfarahin A.J., Soleimani A.F., Kumari S., Aryani A.A., O‘Reilly E.L., Eckersall P.D. (2014). Acute phase proteins, interleukin 6, and heat shock protein 70 in broiler chickens administered with corticosterone. Poult. Sci..

[78] Sohail M.U., Hume M.E., Byrd J.A., Nisbet D.J., Ijaz A., Sohail A., Shabbir M.Z., Rehman H. (2012). Effect of supplementation of prebiotic mannan-oligosaccharides and probiotic mixture on growth performance of broilers subjected to chronic heat stress. Poult. Sci..

[79] Al-Aqil A., Zulkifli I. (2009). Changes in heat shock protein 70 expression and blood characteristics in transported broiler chickens as affected by housing and early age feed restriction. Poult. Sci..

[80] Zulkifli I., Al-Aqil A., Omar A.R., Sazili A.Q., Rajion M.A. (2009). Crating and heat stress influence blood parameters and heat shock protein 70 expression in broiler chickens showing short or long tonic immobility reactions. Poult. Sci..

[81] Soleimani A.F., Zulkifli I., Omar A.R., Raha A.R. (2011). Neonatal feed restriction modulates circulating levels of corticosterone and expression of glucocorticoid receptor and heat shock protein 70 in aged Japanese quail exposed to acute heat stress. Poult. Sci..

[82] Malago J.J., Koninkx J.F., van Dijk J.E. (2002). The heat shock response and cytoprotection of the intestinal epithelium. Cell Stress Chaperones.

[83] Soleimani A.F., Zulkifli I., Hair-Bejo M., Omar A.R., Raha A.R. (2012). The role of heat shock protein 70 in resistance to *Salmonella enteritidis* in broiler chickens subjected to neonatal feed restriction and thermal stress. Poult. Sci..

[84] Heydari A.R., Wu B., Takahashi R., Strong R., Richardson A. (1993). Expression of heat shock protein 70 is altered by age and diet at the level of transcription. Mol. Cell. Biol..

[85] Yu J., Bao E. (2008). Effect of acute heat stress on heat shock protein 70 and its corresponding mRNA expression in the heart, liver, and kidney of broilers. Asian-Australas. J. Anim. Sci..

[86] Soleimani A.F., Zulkifli I., Omar A.R., Raha A.R. (2012). The relationship between adrenocortical function and Hsp70 expression in socially isolated Japanese quail. Comp. Biochem. Physiol. Part A Mol. Integr. Physiol..

[87] Yan J., Bao E., Yu J. (2009). Heat shock protein 60 expression in heart, liver and kidney of broilers exposed to high temperature. Res. Vet. Sci..

[88] Kareem K.Y. (2016). Effect of Postbiotic and Inulin Supplements on Growth Performance, Gut Morthology, Gene Expression and Fecal Characteristics of Broiler Chickens. Ph.D. Thesis.

[89] Thu T., Chwen L., Foo H., Halimatun Y., Bejo M. (2010). Effects of metabolite combinations produced by *Lactobacillus plantarum* on plasma cholesterol and fatty acids in piglets. Am. J. Anim. Vet. Sci..

[90] Pereira D.I., Gibson G.R. (2002). Cholesterol assimilation by Lactic acid bacteria and *Bifidobacteria* isolated from the human gut. Appl. Environ. Microbiol..

[91] Noh D.O., Kim S.H., Gilliland S.E. (1997). Incorporation of cholesterol into the cellular membrane of *Lactobacillus acidophilus* ATCC 43121. J. Dairy Sci..

[92] Den Hertog-Meischke M.J., van Laack R.J., Smulders F.J. (1997). The water-holding capacity of fresh meat. Vet. Q..

[93] Sandercock D.A., Hunter R.R., Nute G.R., Mitchell M.A., Hocking P.M. (2001). Acute heat stress-induced alterations in blood acid-base status and skeletal muscle membrane integrity in broiler chickens at two ages: Implications for meat quality. Poult. Sci..

[94] Liu H.W., Li K., Zhao J.S., Deng W. (2018). Effects of chestnut tannins on intestinal morphology, barrier function, pro-inflammatory cytokine expression, microflora and antioxidant capacity in heat-stressed broilers. J. Anim. Physiol. Anim. Nutr..

[95] Wang R.H., Liang R.R., Lin H., Zhu L.X., Zhang Y.M., Mao Y.W., Dong P.C., Niu L.B., Zhang M.H., Luo X. (2017). Effect of acute heat stress and slaughter processing on poultry meat quality and postmortem carbohydrate metabolism. Poult. Sci..

[96] Zhang Z.Y., Jia G.Q., Zuo J.J., Zhang Y., Lei J., Ren L., Feng D.Y. (2012). Effects of constant and cyclic heat stress on muscle metabolism and meat quality of broiler breast fillet and thigh meat. Poult. Sci..

[97] Zaboli G., Huang X., Feng X., Ahn D.U. (2019). How can heat stress affect chicken meat quality—A review?. Poult. Sci..

[98] Zhou X., Wang Y., Gu Q., Li W. (2010). Effect of dietary probiotic, *Bacillus coagulans*, on growth performance, chemical composition, and meat quality of Guangxi Yellow chicken. Poult. Sci..

[99] Ali F.H. (2010). Probiotics feed supplement to improve quality of broiler chicken carcasses. World J. Dairy Food Sci..

[100] Zheng A., Luo J., Meng K., Li J., Zhang S., Li K., Liu G., Cai H., Bryden W.L., Yao B. (2014). Proteome changes underpin improved meat quality and yield of chickens (*Gallus gallus*) fed the probiotic *Enterococcus faecium*. BMC Genom..

[101] Alvarado C.Z., Richards M.P., O‘Keefe S.F., Wang H. (2007). The effect of blood removal on oxidation and shelf life of broiler breast meat. Poult. Sci..

[102] Petracci M., Betti M., Bianchi M., Cavani C. (2004). Color variation and characterization of broiler breast meat during processing in Italy. Poult. Sci..

[103] Castellini C., Mugnai C., Dal Bosco A. (2002). Effect of organic production system on broiler carcass and meat quality. Meat Sci..

[104] Karaoglu M., Aksu M., Esenbuga N., Kaya M., Macit M., Durdag H. (2004). Effect of dietary probiotic on the pH and colour characteristics of carcasses, breast fillets and drumsticks of broilers. Anim. Sci..

[105] Aksu M., Esenbuga N., Macit M. (2006). pH and colour characteristics of carcasses of broilers fed with dietary probiotics and slaughtered at different ages. Asian-Australas. J. Anim. Sci..

[106] Shimokomakillal A.O.M. (2012). The effects of biotic additives on growth performance and meat qualities in broiler chickens. Int. J. Poult. Sci..

[107] Hao Y., Gu X. (2014). Effects of heat shock protein 90 expression on pectoralis major oxidation in broilers exposed to acute heat stress. Poult. Sci..

[108] Lu Q., Wen J., Zhang H. (2007). Effect of chronic heat exposure on fat deposition and meat quality in two genetic types of chicken. Poult. Sci..

[109] Feng J., Zhang M., Zheng S., Xie P., Ma A. (2008). Effects of high temperature on multiple parameters of broilers in vitro and in vivo. Poult. Sci..

[110] Sato H., Takahashi T., Sumitani K., Takatsu H., Urano S. (2010). Glucocorticoid Generates ROS to Induce Oxidative Injury in the Hippocampus, Leading to Impairment of Cognitive Function of Rats. J. Clin. Biochem. Nutr..

